# Innovative Imaging Technique for Visualization of Vascularization and Established Methods for Detection of Musculoskeletal Inflammation in Psoriasis Patients

**DOI:** 10.3389/fmed.2020.00468

**Published:** 2020-09-02

**Authors:** Michaela Köhm, Lukas Zerweck, Phuong-Ha Ngyuen, Harald Burkhardt, Frank Behrens

**Affiliations:** ^1^Division of Rheumatology, Goethe-University Frankfurt, Frankfurt, Germany; ^2^Clinical Research, Fraunhofer Institute for Molecular Biology and Applied Ecology IME, Branch for Translational Medicine and Pharmacology TMP, Frankfurt, Germany; ^3^Fraunhofer Cluster of Excellence Immune-Mediated Diseases CIMD, Frankfurt, Germany; ^4^Fraunhofer Institute for Applied Information Technology FIT, St. Augustin, Germany; ^5^Centre of Innovative Diagnostics and Therapeutics Rheumatology/Immunology CIRI, Frankfurt, Germany

**Keywords:** psoriasis, psoriatic arthritis, early detection, imaging, musculoskeletal inflammation

## Abstract

Psoriasis (PsO) is one of the common chronic inflammatory skin diseases. Approximately 3% of the European Caucasian population is affected. Psoriatic arthritis (PsA) is a chronic immune-mediated disease associated with PsO characterized by distinct musculoskeletal inflammation. Due to its heterogeneous clinical manifestations (e.g., oligo- or polyarthritis, enthesitis, dactylitis, and axial inflammation), early diagnosis of PsA is often difficult and delayed. Approximately 30% of PsO patients will develop PsA. The responsible triggers for the transition from PsO only to PsA are currently unclear, and the impacts of different factors (e.g., genetic, environmental) on disease development are currently discussed. There is a high medical need, recently unmet, to specifically detect those patients with an increased risk for the development of clinically evident PsA early to initiate sufficient treatment to inhibit disease progression and avoid structural damage and loss of function or even intercept disease development. Increased neoangiogenesis and enthesial inflammation are hypothesized to be early pathological findings in PsO patients with PsA development. Different disease states describe the transition from PsO to PsA. Two of those phases are of value for early detection of PsA at-risk patients to prevent later development of PsA as changes in biomarker profiles are detectable: the subclinical phase (soluble and imaging biomarkers detectable, no clinical symptoms) and the prodromal phase (imaging biomarkers detectable, unspecific musculoskeletal symptoms such as arthralgia and fatigue). To target the unmet need for early detection of this at-risk population and to identify the subgroup of patients who will transition from PsO to PsA, imaging plays an important role in characterizing patients precisely. Imaging techniques such as ultrasound (US), magnetic resonance imaging (MRI), and computerized tomography (CT) are advanced techniques to detect sensitively inflammatory changes or changes in bone structure. With the use of these techniques, anatomic structures involved in inflammatory processes can be identified. These techniques are complemented by fluorescence optical imaging as a sensitive method for detection of changes in vascularization, especially in longitudinal measures. Moreover, high-resolution peripheral quantitative CT (HR-pQCT) and dynamic contrast-enhanced MRI (DCE-MRI) may give the advantage to identify PsA-related early characteristics in PsO patients reflecting transition phases of the disease.

## Introduction

Psoriasis (PsO) is one of the common chronic inflammatory skin diseases. Approximately 3% of the European Caucasian population is affected. Psoriatic arthritis (PsA) is a chronic immune-mediated disease associated with PsO characterized by distinct musculoskeletal inflammation. Due to its heterogeneous clinical manifestations (e.g., oligo- or polyarthritis, enthesitis, dactylitis, and axial inflammation), and the fact that only classification criteria are available [ClASsification for Psoriatic ARthritis (CASPAR) criteria], early diagnosis of PsA is often difficult to set and frequently delayed. Approximately 30% of PsO patients will develop PsA in their lifetime. Moreover, many patients develop a destructive form of arthritis with substantial morbidity and disability (1). Reasons for the transition from PsO to PsA are currently unclear as well as their impact on disease development. It is currently discussed that different factors such as genetic and/or clinical–demographic risk factors (e.g., nail PsO, PsO severity and type) may promote PsA development and its progression. Risk factors are observed in different epidemiologic studies, but there seems to be as well a heterogeneous profile for different factors identified (2).

The underlying molecular mechanisms for the transition from PsO to PsA are still poorly defined but better understood due to the use of biologic treatments addressing different target molecules for both PsO and PsA. Similar to the predictive value of anti-citrullinated peptide antibodies (ACPAs) for the development of rheumatoid arthritis (RA) in patients with arthralgia, the medical condition of PsO as an underlying disease describes an at-risk population to develop PsA. There is a high medical need, recently unmet, to identify early those patients with an increased risk for PsA development and first signs of musculoskeletal inflammatory changes for clinically evident PsA development. Only by early detection, sufficient treatments to inhibit disease progression and avoid structural damage and loss of function can be initiated. Moreover, here, the interception of PsA may be targeted with sufficient treatment when initiated in a very early stage of the PsO patients at risk for PsA development (principle of disease interception). However, ~50% of patients with PsO present with subclinical imaging enthesopathy, and only a subgroup of them will develop PsA (3). So, even as the evidence of signs for musculoskeletal inflammation being found by use of sensitive imaging technique may be of high value to identify the at-risk collective, the technique must also be appropriate to differentiate between disease-related changes of PsA to predict progression from PsO to clinically manifested PsA. Therefore, there is a need for a specific biomarker panel including either soluble molecular markers and/or sensitive imaging markers to solve this challenge. Different imaging techniques are available with various modes to identify inflammation or inflammatory changes and heterogeneous impact for sensitive detection of signs for very early musculoskeletal inflammatory changes.

## The Transition From Psoriasis To Psoriatic Arthritis

PsA mostly develops in patients with established PsO; only in 15% of patients PsO occurs in parallel or after PsA development (4). The incidence of PsA with PsO onset increases with time, reaching ~20% after 30 years (5). Nail, scalp, and inverse PsO and its severity are identified as potential clinical factors for increased risk for PsA development (4). In focusing on comorbidities, obesity is identified as a strong risk factor for PsA development (6), with an additional link of the magnitude of body mass index (BMI) and PsA risk (7). For genetic background, a first-degree relative with arthritis contributes to the risk (8). Moreover, arthralgia mainly in female PsO patients is a strong predictor for PsA development (9). This finding was confirmed by Zabotti et al. (10) in 2019 in a longitudinal study.

**Figure 1:**
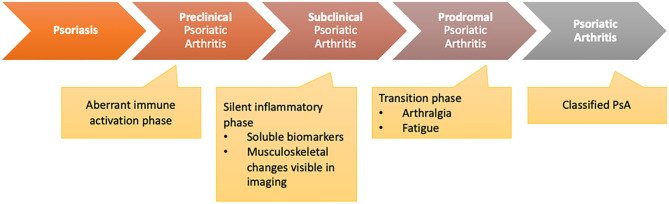
Transition model from psoriasis to psoriatic arthritis [adapted from Scher et al. (2)].

Scher et al. (2) recently proposed three clinically inconspicuous stages after PsO onset and before the clinical presentation of PsA: (a) a preclinical phase characterized by aberrant activation of the immune system which may originate from the skin, intestinal mucosa, or the entheses; (b) the subclinical PsA phase with the detection of soluble biomarkers and imaging findings without clinical symptoms; (c) the prodromal phase of PsA in which patients report unspecific symptoms such as arthralgia and fatigue without objective detection of signs of musculoskeletal inflammation with synovitis and/or enthesitis in physical examination (Figure 1).

So, the subclinical and prodromal phases are linked by changes that might be detectable by established biomarkers or a specific biomarker profile including imaging techniques predicting later clinical development of PsA. In the clinical care setting, patients will mainly be presented to a specialist rheumatologist in the prodromal phase, when clinical symptoms such as arthralgia and fatigue occur. The subclinical identification of PsA will be necessary for dermatologists or general practitioners (GPs) to select the patients being presented to the rheumatologist or change treatment strategy.

In both scenarios, the biomarker to detect either patient in the subclinical or prodromal phase must be sensitive for detection of musculoskeletal inflammatory changes but also specific to identify those changes as PsA-related and highly predictive for later development of clinically evident PsA. Imaging studies of early PsA suggest that inflammatory enthesopathy is an early sign that is presented in PsA development (11). However, as shown in longitudinal studies, ~50% of patients with PsO that present with subclinical imaging enthesopathy will develop clinically manifested PsA (3).

## Imaging Techniques for The Detection of Early Changes In Angiogenesis and Musculoskeletal Inflammation

Different imaging techniques are available in clinical routine care to determine inflammatory musculoskeletal changes and increase in angiogenesis (Table 1). Different vascular and musculoskeletal compartments can be assessed and rated in focus on changes in vascularization, the severity of inflammation, disease manifestation, and disease state. Fluorescence optical imaging (FOI) is an indocyanine green (ICG)-tailored imaging technique that visualizes changes in micro-vascularization of the hands. FOI is a well-tolerated and fast method that seems sensitive for the detection of signs of inflammation and their changes over time. Nevertheless, it is limited by the rate of skin texture responsible for the penetration depth of the measurement system. Imaging of bone structure, entheses, and synovia is of high importance for classification of disease state and disease activity. By use of ultrasonography (US), changes in synovial and enthesial structure in early inflammation can be detected as it is commonly used but restricted by the experience of the observer and assessment algorithm. Moreover, by the use of US, changes of vascularization in the synovia and the enthesial structures can be easily evaluated using Doppler mode presenting as a sensitive marker for inflammation. Magnetic resonance imaging (MRI) is used to assess the presence of bone marrow edema, enthesitis, and changes in vascularization as early and disease activity indicators of inflammation of the joints, entheses, and spine. X-ray and computerized tomography (CT) can be used to assess structural damage in two and three dimensions. Innovative methods, such as high-resolution peripheral quantitative CT (HR-pQCT), can visualize pathophysiological processes and the morphological consequences at the bones even in the early stages of the disease. Moreover, techniques such as dual-energy CT (DECT) with iodine mapping may be helpful in discrimination of early arthritis.

**Table 1:** Overview of imaging techniques available for diagnosis of psoriatic arthritis (PsA) in psoriasis (PsO) patients and their advantages and disadvantages.

**Technique**	**Advantage**	**Disadvantage**
X-Ray	• Bone structure can be assessed (late changes)	• (Availability) • Radiation • Sensitivity for early changes: only structural changes to be detected • Only one structure per examination
Computerized tomography (CT)	• Bone structure can be assessed in 3 dimensions and related to anatomically structures (late changes)	• Availability • Sensitivity for early changes: only structural changes to be detected • Radiation • Only one structure per examination
High-resolution peripheral quantitative CT (HR-pQCT)	• Sensitivity: early structural changes can be detected	• Availability • Radiation • Only one structure per examination
Scintigraphy	• Availability • Whole body scan for increased metabolism (related to inflammation)	• Application of radioactive tracer • Poor specificity
^18^F-FDG PET	• Whole body scan for increased metabolism (related to inflammation)	• Availability • Tolerability • Poor specificity
Ultrasound (US)	• Availability • Tolerability • Scan of all anatomically related structures possible • Doppler function: increase in vascularisation	• Time consuming when used in all joints and msk structures • Rater dependent (experience)
Magnetic Resonance Imaging (MRI)	• Scan of all anatomically related structures possible • Bone structure and metabolism	• Availability • Tolerability (contrast agent) • Only one structure per examination
Whole Body MRI (WB-MRI)	• Scan of whole body in one examination • Bone structure and metabolism	• Availability • Tolerability (contrast agent)
Dynamic contrast enhanced MRI (DCE-MRI)	• Scan of all anatomically related structures possible • Bone structure and metabolism • Quantification of inflammation	• Availability • Tolerability (contrast agent) • Only one structure per examination
Fluorescence-optical imaging (FOI)	• Availability • Tolerability • displays changes in micro vascularisation of the hands	• Only hands • Depth of measure limited • Rater dependent for (maunal) assessment

### Fluorescence Optical Imaging

**Figure 2:**
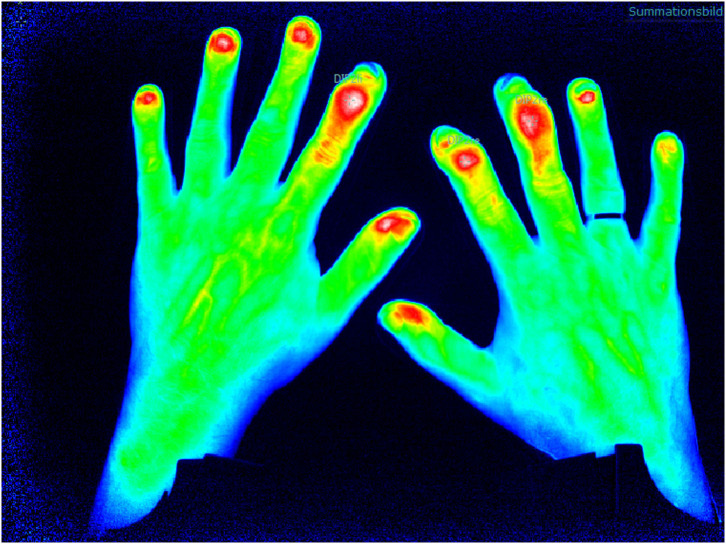
Fluorescence optical imaging (FOI) assessment with detection of distal interphalangeal (DIP) arthritis.

Increase in micro-vascularization is identified as an early marker for PsA and of higher value than in other inflammatory joint diseases such as RA and may be a parameter to distinguish PsO from PsA in the subclinical phase of transition (12). FOI is an innovative imaging technique which detects changes in micro-vascularization of the hands as the camera system is only available for the hands by now. The method is tailored to indocyanine green injection which is a tolerable color agent used in different medical indication fields. The assessment lasts 360 s (one picture per second), and after measurement, the ICG kinetic profile is used for the (manual) assessment (Figure 2).

First studies to validate FOI were performed by assessment of its ability to detect synovitis/arthritis compared to the US and MRI examination (sensitivity 76%, specificity 94%) (13). Thuermel et al. (14) compared different disease states of synovitis between FOI and MRI findings. It was shown that mild synovitis was discriminated poorly by both FOI and MRI (81.6 vs. 86.8%), but with worsening of severity of synovitis, the potential for the discriminative ability increased in both methods with high correlation (moderate synovitis false positive in 12.5 vs. 16% for FOI and severe synovitis false positive in 0.7 vs. 2.4% for FOI). Additionally, Hirano et al. (15) confirmed the correlation of FOI measurement in the assessment of synovitis compared to US examination.

**Figure 3:**
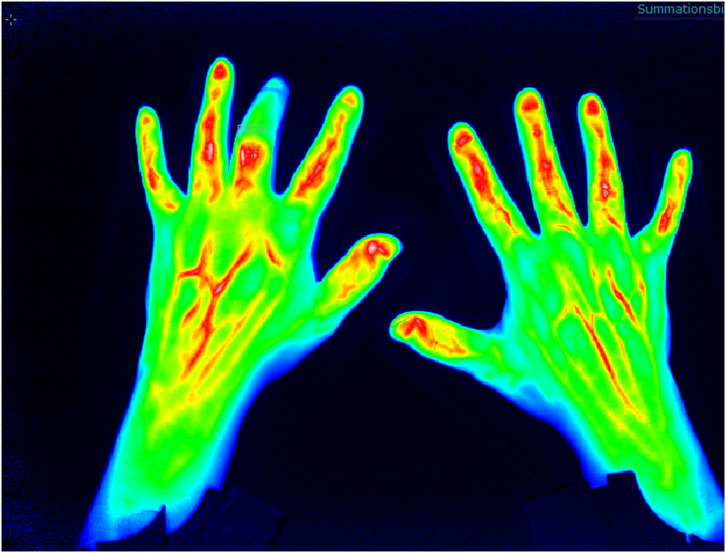
Fluorescence optical imaging (FOI) assessment of arthritis/tendinitis in the hands.

The different morphologic patterns can be identified in PsA patients that might be specific for the disease (Figure 3): in 94% of patients with clinically manifested PsA, a triangular increase of the color agent is detected at distal interphalangeal (DIP) joints (compared to 21% of RA patients), in 21% of PsA patients, a “green nail” sign was detectable (compared to 3% in RA patients), with a specificity of 87% and a sensitivity of 28% (16).

In the XCITING study, FOI was performed in patients with PsO and at risk for PsA development (nail PsO, musculoskeletal complaints within the last 6 months). In ~46% of the patients, FOI showed an increase of micro-vascularization defined as related to subclinical PsA (with the determination of inflammatory changes in MRI in 37%). Here, longitudinal data will show how many of those patients develop PsA in a 24-month follow-up period (17) to indicate its potential to select the at-risk population of PsO patients with later overt PsA. Therefore, FOI might be a promising imaging technique with little limitations (injection of the tolerable color agent, manual reading with the need of an experienced reader) and high advantages (fast performance, high sensitivity) to be of value in early detection in the transition from PsO to PsA.

#### Objective Joint Evaluation Based on Fluorescence Optical Imaging

FOI is assessed with the use of a manual assessment algorithm published by Werner et al. (13). To use FOI for the screening of PsO patients for changes in vascularization suspect for PsA, an objective assessment method is needed to allow fast assessment of the images with an automated algorithm. Therefore, methods to automate the assessment of the fluorescence intensities are in development.

To visualize the micro-vascularization of the hands with the FOI technique, a time-dependent data set of 360 images is acquired. Each image shows the current state of the proceeding distribution of the contrast agent ICG. To objectively evaluate the status of the subject's joints individually, the micro-vascularization at each joint position is evaluated.

**Figure 4:**
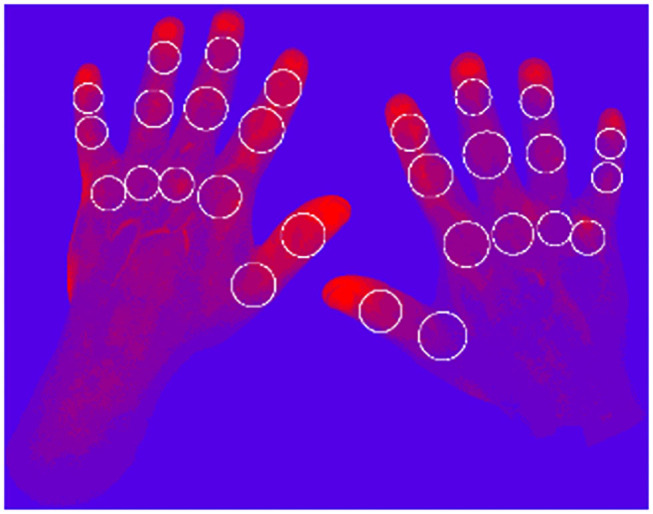
Example: Nine clusters, maximum slope.

To represent the status of each joint, several scores are calculated, representing different characteristics of the micro-vascularization. In the first step, each pixel (680 512) is represented by a time series containing 360 values. For each time series, three different features are extracted: the amplitude, the mean value during the signal increasing time, and the maximal gradient. Then, each of the three sets is clustered by k-means clustering, with *k* ∈ {3, 5, 7, 9}. Furthermore, each pixel is assigned a shade of red–blue, where the brightest (red) corresponds to the highest cluster indicating highest dynamics and the darkest to the lowest indicating the smallest dynamics (Figure 4).

Therefore, 12 heatmaps (three features 4 *k*s) are generated. Finally, scores for the defined joint areas are calculated based on the assigned cluster values, number of pixels in the joint area and the number of clusters *k*.

Applying this newly developed method to 271 clinical examination (CE) labeled patients with 6,426 healthy joints and 1,162 affected joints (tender, swollen, or both) results in a clear distinction between the scores for healthy and affected labeled joints. The summarized results for *k* = 9 are visualized in Table 2.

**Table 2:** Resulting scores for *k* = 9 for all 271 patients.

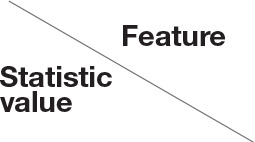	**Amplitude**		**Mean**		**Slope**	
	**Healthy**	**Affected**	**Healthy**	**Affected**	**Healthy**	**Affected**
Average	0.503	0.528	0.486	0.509	0.395	0.414
Median	0.496	0.532	0.482	0.505	0.389	0.415

### Ultrasound

**Figure 5:**
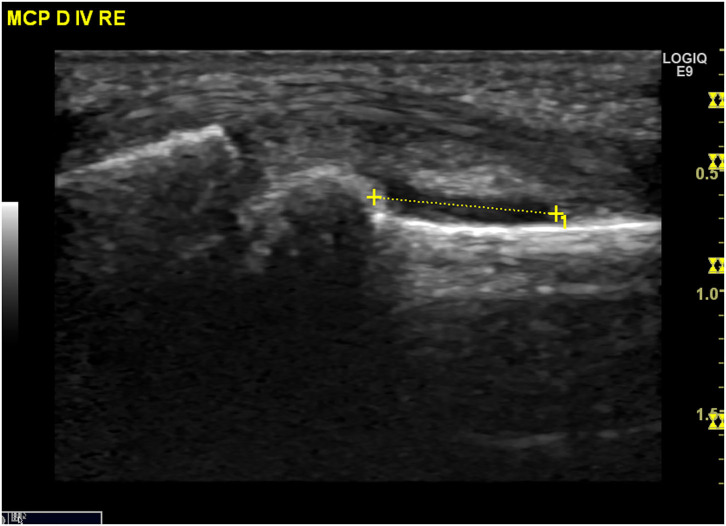
Ultrasound assessment of synovitis metacarpophalangeal joint digit IV (MCP D IV).

**Figure 6:**
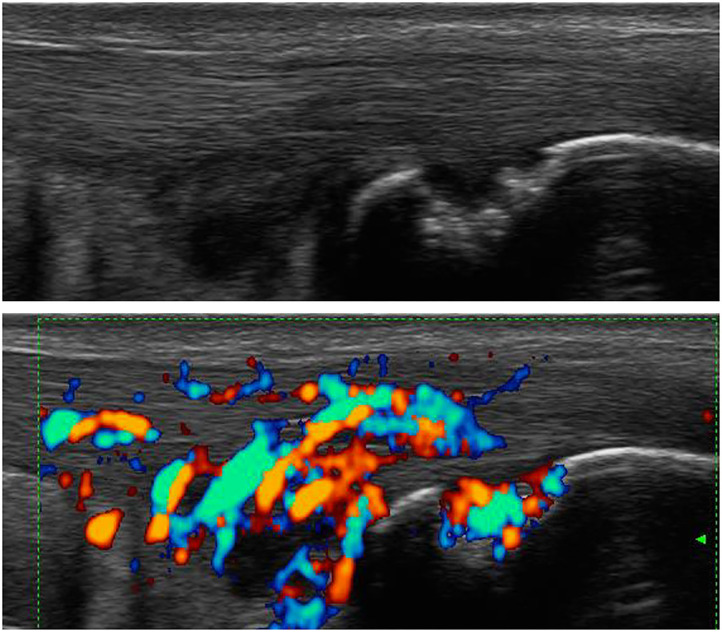
Ultrasound assessment. Tendinitis of the Achilles tendon with the formation of osteophytes and power Doppler activity.

**Figure 7:**
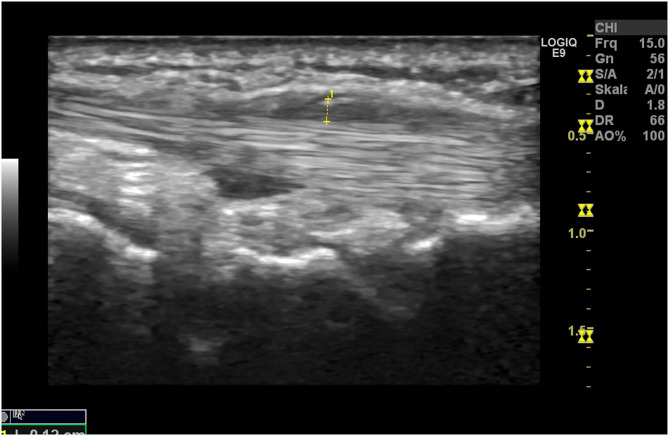
Ultrasound assessment of flexor tendinitis.

Ultrasound (US) is used to identify structural and inflammatory changes in the joints and the musculoskeletal system (Figures 5–7). In different clinical studies, it has been demonstrated that US examination is more sensitive than radiography (18). The US is more sensitive than clinical examination for the assessment of inflammatory and structural changes in inflammatory arthritis, including PsA, and particularly synovitis, enthesitis, tenosynovitis, and bursitis (19, 20). Besides, US results are comparable to those of MRI, except for the detection of bone marrow edema. The reproducibility and low cost of US examination provide advantages compared to MRI for early PsA detection (21). However, a complete US examination of all joints, entheses, tendons, and bursae is extremely time-consuming and infeasible. Moreover, US examination is assessor related and depends on the experience of the rater.

As it was shown that inflammatory enthesopathy is a subclinical sign in the transition from PsO to PsA, the US may be a sensitive and feasible method to detect early inflammatory changes at entheses. Savage et al. (22) showed that subclinical enthesopathy and associated osteitis are present in patients with PsO but without arthritis, for which clinical examination is ineffective. And 49.3% of the PsO patients included had no evidence of clinically manifested PsA but showed at least one sonographic inflammatory abnormality that fulfilled the OMERACT definition of enthesopathy. These findings support the assumption that even in PsO, changes at the enthesial sites are detectable. It is necessary to assess in longitudinal how many of those patients developed overt PsA to specify these findings for risk stratification.

### Magnetic Resonance Imaging

**Figure 8:**
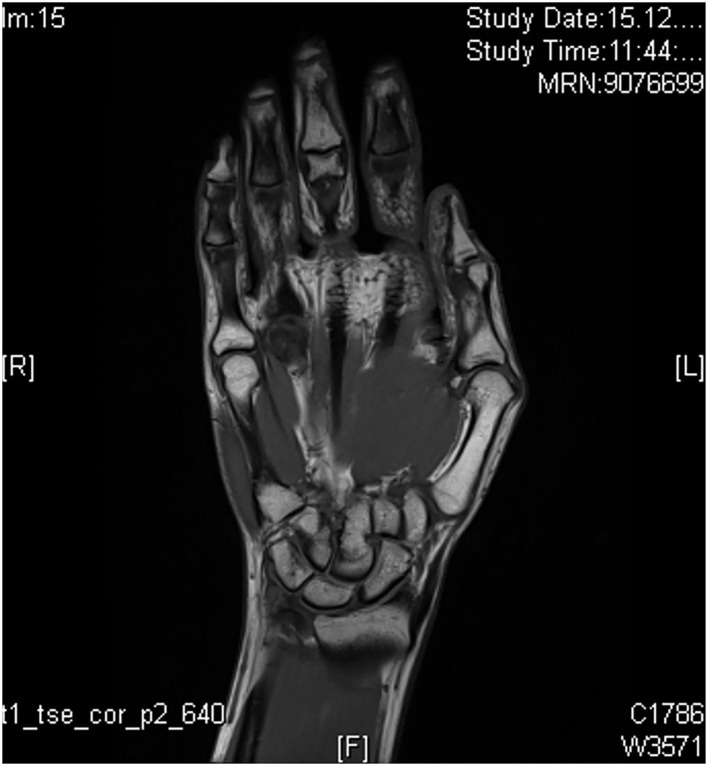
MRI of the left hand without signs of inflammation (T1 sequence).

MRI is a very sensitive method for visualization of musculoskeletal structures that are involved in inflammatory processes. There are only little data on the detection of early PsA as the main studies on the detection of early arthritis were performed for the indication field of RA (23). With the use of MRI, arthritis, synovitis, tenosynovitis, periarticular inflammation, bone marrow edema, erosions, and bone proliferation can be sensitively visualized and anatomically classified. Limitations of MRI examination are the need of long examination times with illustration of only a single anatomic structure per examination and the need of contrast agent (tolerability). Definitions of disease-specific abnormalities are provided by the OMERACT working group, whereas enthesial disease criteria are only recently published (24). For inflammation and structural changes, T1-weighted sequences in two planes are performed (signal mainly reflecting fat content and a contrast agent), supplemented with a T2-weighted, fat-suppressed sequence or short tau inversion recovery (STIR) sequence (signal mainly reflecting water content). Performance of additional T1-weighted sequences (after intravenous gadolinium-containing contrast agent; Figure 8) with or without fat suppression assists in the assessment of tissue inflammation in peripheral joints. Use of contrast agent is needed for illustration of synovitis and tenosynovitis but not for detection of erosions, bony proliferation, and bone marrow edema (24).

Differences between PsA and other inflammatory diseases classified by MRI are reported. In PsA, enthesitis is not related to a focal inflammation but to a wider “synovio-enthesal complex” (25), including adjacent tendons, periosteum, fibrocartilage, synovium, and bone at the attachment sites. Whereas, MRI findings such as synovitis, enthesitis, tenosynovitis, and bone marrow edema that appear frequently in PsA are not disease-specific and appear in any other inflammatory joint disease, PsA is characterized by more prevalent diaphyseal bone marrow and/or enthesitis, soft tissue inflammation, extracapsular inflammation, and involvement of primarily flexor tendons in contrast to extensor tendons in RA (26). Erosions are often located close to the collateral ligaments in contrast to osteoarthritis where they are frequently found centrally (27). In comparison to RA, in PsA, periostitis is found more frequent than erosions (28).

Assessments in PsO patients without evident arthritis revealed subclinical inflammation in both joints and entheses. Moreover, it was found that PsO patients with subclinical inflammation in MRI and arthralgia had a high risk (55.5%) for the later development of PsA, whereas patients without arthralgia had a low risk (15.3%) for PsA development (29). The specific pattern is recently not defined to detect PsO patients in subclinical development of PsA.

New methods of MRI such as whole-body MRI (WB-MRI) and dynamic contrast-enhanced MRI (DCE-MRI) are used in the research of PsO and PsA discrimination and prediction of PsA development. DCE-MRI allows semiautomated quantification of inflammation based on the measurement of contrast enhancement pattern over time in the selected region of interest. A recent report reports differences in RA and PsA in the pattern of enhancement of the synovial membrane with higher inflamed tissue in RA but a higher degree of inflammation in PsA (30). WB-MRI assessment indicated agreement between enthesitis on clinical scores and MRI results in PsA patients, suggesting value in detecting subclinical inflammation and the total burden of inflammation (31, 32).

### Conventional X-Ray and Computerized Tomography

**Figure 9:**
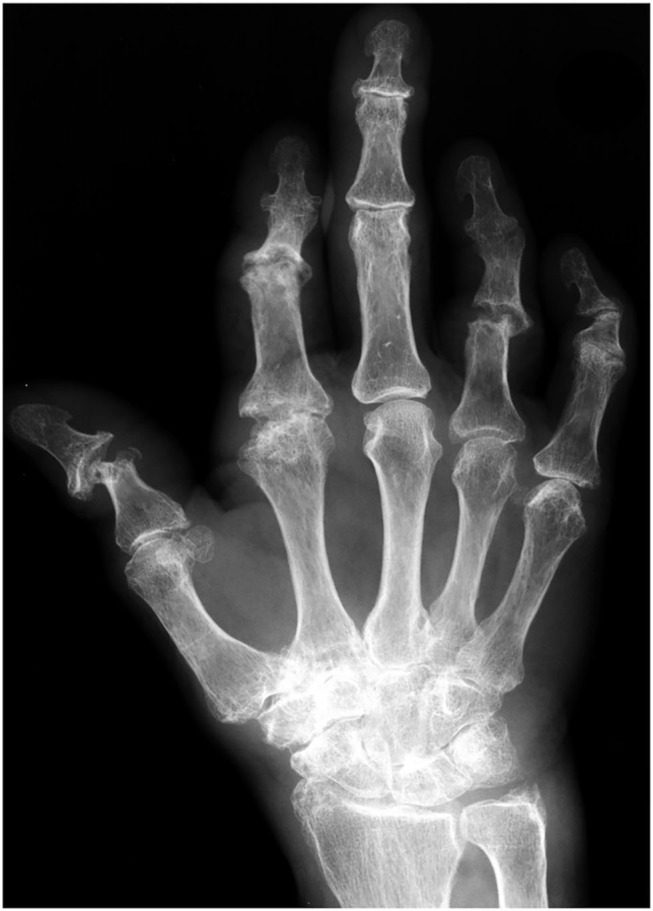
Plain x-ray of the right hand with arthritis mutilans.

Conventional radiographic imaging methods such as x-ray (Figure 9) or conventional CT are often used to detect changes in bone structure in progressive PsA after clinical detection of inflammation by assessment of swollen or tender joints (33). The sensitivity for the detection of early changes in PsO patients at risk for PsA development is poor as only pronounced changes in bone structure are detectable being evident and often found only in a late stage of the disease.

### Innovative Techniques Using Computerized Tomography Methods

Innovative methods, such as high-resolution peripheral quantitative CT (HR-pQCT) are available to sensitively detect early changes in bone metabolism in PsO patients compared to PsA and other joint diseases. Bone erosions can be detected early in the disease state of PsA (34), and the severity of erosions depends on its articular type and its activity (inflammation) status (35). Bone erosions in PsA pathophysiology result from an accumulation of osteoclasts in the joints, which is promoted by pro-inflammatory cytokines (36). Anabolic bone changes in PsA are mainly based on new bone formation. These changes typically occur at insertion sites of tendons to bone. Enthesial inflammation is a key process in PsA and most likely triggered by mechanical stress (37). Bone structure responses at the inflamed enthesial sites result in the formation of enthesophytes. An HR-pQCT-based study showed that the formation of enthesophytes are pronounced in PsA but are not found in rheumatoid arthritis (38). Furthermore, a more recent study revealed that enthesophytes occur early in patients with PsO without joint involvement, suggesting that their formation reflects a common process in PsO and PsA (39). In another study from Simon et al. (40), bone structure, number erosions, and enthesophytes were compared between different patient cohorts (PsO, PsA, and healthy controls) to measure a discriminative potential. Data on the extent of bone erosions and enthesophytes were collected and correlated to different categories of age, duration of PsO, and duration of PsA. Additionally, demographic and disease-specific data, including physical function [Health Assessment Questionnaire (HAQ)], were collected. A total of 203 patients were analyzed (101 with PsA, 55 with PsO, and 47 as healthy controls). Patients with PsA had a significantly higher number with a higher extent of erosions and enthesophytes compared to patients with PsO and healthy controls. Patients with PsO and healthy controls did not differ in number and extent of erosions, while enthesophytes were more frequent in patients with PsO than in healthy controls. Bone erosions, but not enthesophytes, showed strong age dependency in all three groups. In contrast, enthesophytes were mostly influenced by the duration of PsO and PsA and, in contrast to bone erosions, were associated with poorer physical function, as measured by HAQ. Unfortunately, data on the association between the risk collective of PsO patients who develop PsA are currently missing but under examination. The longitudinal change will give more insights into the potential of HR-pQCT as an imaging method predicting PsA development in PsO patients.

Besides HR-pQCT, dual-energy CT (DECT) with iodine mapping to improve iodine contrast resolution may be a sensitive method to discriminate early inflammatory arthritis (41) but restricted in longitudinal measurement by radiation rate. Only a few studies were performed by now showing potential for early discrimination, but further studies are needed to confirm the findings in larger cohorts and over time.

### Scintigraphy

Bone scintigraphy is an established method displaying an increase of metabolism of the tracer as a sign for abnormal metabolism rates, e.g., in the inflammatory state. It is used to detect inflammatory changes in the whole body, especially if other imaging techniques are not available. It has poor specificity and is limited by the use of the radioactive isotopes (tracers). In PsA, it was used in different cohorts to detect subclinical signs of inflammation in PsO patients without evident clinical PsA. The results were compared to the findings in US examination and clinical examination (42). It was revealed that a significantly higher number of joints were verified by the US to have increased uptake of tracer compared to clinical examination (34).

### Fluorine-18-Labeled Fluorodeoxyglucose Positron Emission Tomography

Fluorine-18-labeled fluorodeoxyglucose positron emission tomography (^18^F-FDG-PET) is a very sensitive imaging method for detection of changes in metabolism, e.g., inflammation-dependent early musculoskeletal changes indicating PsA-related changes. Takata et al. (43) performed a clinical study in PsO patients without clinically manifested PsA compared to PsA patients with long-lasting clinical overt PsA. Eighteen PsO and 28 PsA patients were enrolled for examination by positron emission tomography/computerized tomography (PET/CT) using ^18^F-FDG. In the PsA cohort, ^18^F-FDG accumulation was identified in all affected joints, illustrating its reliability in correlation to clinical examination sensitivity. In the PsO cohort, asymptomatic enthesitis was detected in six out of 18 PsO patients (33%), demonstrating its high sensitivity in the detection of inflammation at the entheses.

### Imaging Techniques for Sensitive and Specific Detection of Early Psoriatic Arthritis In Different Transition Phases

**Figure 10:**
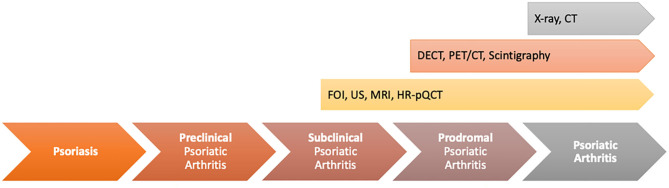
Value of different imaging techniques to find unspecific musculoskeletal changes in psoriasis (PsO) patients with the transition phases from PsO to psoriatic arthritis (PsA).

The transition from PsO to PsA contains different phases (preclinical, subclinical, and prodromal). In only two of those phases, early changes in soluble biomarkers/imaging can be detected (subclinical phase) or first unspecific clinical symptoms (e.g., arthralgia and fatigue) occur (prodromal phase). These two phases may be useful in early discrimination of the PsO patients at risk to develop overt PsA to prevent its occurrence (Figure 10).

In the transition model, the subclinical phase includes early musculoskeletal inflammatory changes in PsO patients without musculoskeletal complaints. In this phase of the disease, MRI, HR-pQCT, and high-frequency ultrasonography might have the highest potential to distinguish inflammatory changes in the defined at-risk-population with the limitation of the specificity to be PsA-related. By a combination of these techniques, patients are deeply characterized in focus on first musculoskeletal inflammatory changes and early changes in bone structure/bone metabolism. This characterization is completed by the use of FOI to generate additional information on early changes in vascularization as signs of increased angiogenesis, reported as an early marker in PsA progress (12). In different clinical studies within this special patient population, it was shown that in a high proportion of asymptomatic PsO patients, enthesial inflammation was detectable (44). Moreover, Simon et al. (40) showed that by the use of HR-pQCT, the number of enthesophytes was significantly higher in asymptomatic PsO patients than in healthy controls. Moreover, the duration of skin disease influenced the number of enthesophytes (45). Nevertheless, only a part of those patients will develop clinical overt PsA afterwards. The potential of imaging biomarker to detect the specific at-risk population is poor. Additionally, clinical definitions adapted from other inflammatory diseases such as the criteria for subclinical inflammation in RA are not usable in PsA as by its use, half of the asymptomatic PsO patients are classified as subclinical PsA, but only part of them will develop PsA later. So, within this phase is a high need of clear classification and characterization of patients to define the correct at-risk population for PsA development, maybe by a combination of different biomarkers (e.g., combination with soluble biomarkers, e.g., on miRNA base, biomarkers for comorbid conditions that were shown to occur early in the disease process or different imaging techniques). Beside the definition of the biomarker set, its usability must be high to be implemented into clinical routine care.

The prodromal phase of the disease is classified as unspecific clinical symptoms such as arthralgia and/or fatigue combined with the occurrence of inflammatory changes in imaging. A recent study showed that, in this phase, tenosynovitis was the most significant contributor to the reported musculoskeletal symptoms (PsO vs. PsO with arthralgia vs. PsA). In the US, in a high proportion of patients, tenosynovitis especially of the flexor tendon of the hands was detected (in 29.5% of the arthralgia patients compared to 5.3% in the PsO group) (10), whereas active enthesitis and synovitis did not reach a significant difference. In the longitudinal part of the study, in the US, determined enthesitis was the only US feature linked to the future evolution of PsA. Faustini et al. (29) confirmed this link between subclinical inflammation detected and PsA, highlighting that patients with synovitis detected by MRI and arthralgia had 55.5% likelihood to develop PsA within 1 year. In the IVEPSA (Interception in very early PsA) study, psoriatic patients with inflammatory arthralgia without joint swelling and with concomitant predictors of PsA [i.e., Psoriasis Area and Severity Index (PASI) >6 or scalp PsO or nail involvement] were treated with anti-interleukin (IL)-17 for a disease interception. Baseline MRI investigation of the dominant hand revealed at least one inflammatory lesion in 83% of patients, highlighting synovitis as the most prevalent (66.7%), followed by tenosynovitis (55.6%) (46).

## Discussion

PsO is one of the common chronic inflammatory skin diseases, affecting ~3% of the European Caucasians. PsA is a chronic immune-mediated disease associated with PsO characterized by distinct musculoskeletal inflammation. Due to its heterogeneous clinical manifestations (e.g., oligo- and polyarthritis, enthesitis, dactylitis, and axial inflammation), early diagnosis of PsA is often difficult and delayed. PsO patients are of high risk for PsA development as ~30% of PsO patients will be affected by (chronic) musculoskeletal inflammation.

The main events for the transition from PsO to PsA are currently unclear: the combination of genetic and clinical–demographic risk factors (e.g., nail PsO, PsO severity, and type) may promote PsA development and its progression. The underlying molecular mechanisms for the transition from PsO to PsA are still poorly defined. The increase of neoangiogenesis (12) and the development of enthesitis is hypothesized to be a primary manifestation in PsA, detectable early using imaging and clinical examination in patients developing later overt PsA (22).

Different phases were defined to describe the transition from PsO to PsA (2). Three phases are named between PsO and overt PsA of which two have high relevance to be detectable in the at-risk population with the use of sensitive biomarkers such as sensitive imaging techniques: the subclinical phase (detection of soluble biomarkers, e.g., on miRNA base and early musculoskeletal inflammation such as synovio-enthesitis detected in imaging but missing related clinical symptoms) and the prodromal phase (detection of musculoskeletal inflammation in imaging techniques and unspecific clinical symptoms such as fatigue and arthralgia).

**Figure 11:**
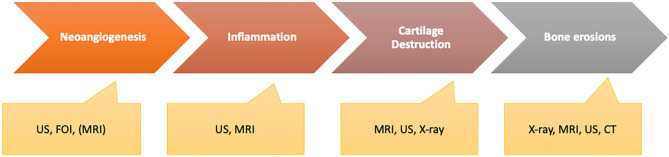
Different disease activity characteristics with the ability of imaging methods to be measured adequately.

The potential of the available imaging techniques to detect patients in one of these two phases differs widely as all of these techniques characterize different pathophysiologic states of the inflammatory process that might have the highest value by being combined for deep characterization of the patients (Figure 11).

Besides sensitivity and specificity in the detection of anatomical and pathophysiological changes by use of advanced imaging techniques, standardized assessment tools are needed to bring innovative imaging methods in context to the suspected indications and process results to enable detection of coherence between saliences and the underlying disease. Technological advances should be integrated into assessment algorithms to use its full potential to be beneficial in the detection of at-risk patients especially in context to PsO and PsA patients.

FOI as an ICG-tailored imaging technique visualizes changes in micro-vascularization of the hands (13). It is a well-tolerated and fast method that seems sensitive for detection of early signs of inflammation related to changes in angiogenesis. Early changes of micro-vascularization may be indicators for early signs of inflammation of the joints and tendons as neoangiogenesis was identified as a symptom associated with musculoskeletal inflammation in PsA differently to other arthritides (47). Due to its mechanism, the detection of changes in micro-vascularization may be of value to define a very early disease state of PsA in the subclinical phase of transition when clinical signs such as arthralgia are missing. With its high sensitivity to detect changes, it is of value to be used in longitudinal observation. These aspects should be investigated in further studies as it gives an advantage to easily screen PsO patients without symptoms but defined risk profile early. Automated measurement of the fluorescence signals gives an objective assessment of the state and its changes over time when used in longitudinal assessments.

MRI and US are the recent widely used methods for sensitive detection of inflammation in the joints and musculoskeletal structures (entheses, bursa, tendons). Bone marrow edema, enthesitis, and changes in vascularization as early indicators of inflammation of the joints, entheses, and spine in the subclinical and prodromal phases can be visualized with high sensitivity by both (23). The US has the benefit of its availability as well as of its possibility to detect changes in synovial and enthesial structure in early inflammation. Changes of vascularization in the synovia and the enthesial structures can be easily evaluated using Doppler mode presenting as a sensitive marker for inflammation (subclinical and prodromal phase) (44). Structures of interest can be associated with anatomica structures, and due to their tolerability, longitudinal status can be measured easily.

HR-pQCT, developed from CT technique, might be of high value to detect early changes in bone metabolism and structure in PsO and PsA patients (compared to other inflammatory diseases), but the value for discrimination of PsO or PsO transient to PsA is recently not clear. Simon et al. (40) showed that in PsO and PsA patients, the formation of enthesophytes is pronounced compared to RA and healthy controls, but the differentiation of those findings between PsO in early PsA state is poor. Longitudinal data are missing to evaluate within this group. Dual-energy CT might be as well a very sensitive method to discriminate between PsO/PsA disease state, but its performance and repeatability are limited to radiation and validity.

Established methods such as x-ray or CT are not sensitive enough to detect early inflammatory changes of the musculoskeletal disease but changes in bone structure.

PET/CT and scintigraphy have established methods with a focus on metabolism and its changes in the inflammatory disease state. Both methods are limited by their poor specificity but sensitive to illustrate changes early and with focus on the whole body not restricted by use in one region of interest.

For US, MRI, and HR-pQCT, the relevance of the early findings of enthesophytes (HR-pQCT) or detection of enthesopathy (MRI, US) as signs for early musculoskeletal inflammation without clinical symptoms (subclinical phase) in PsO patients is still unclear. In a longitudinal study, it was found that although these findings were detectable in 50% of the PsO patients, only a part of them developed overt PsA later in their disease state (40, 45). So, these imaging findings are not specific for the definition of high risk for PsA development and must be completed by a multifactorial characterization of the patient profile, e.g., using data on the comorbid condition, clinical data, and data of soluble biomarker (in the investigation).

The potential of these imaging techniques increases in the prodromal phase when clinical symptoms appear, even if they are non-specific. Here, the at-risk population is enriched, and the combination of both imaging findings and arthralgia predicts the development of PsA significantly.

Overall, different imaging techniques are available with various methods to sensitively quantify vascularization and early signs of musculoskeletal inflammation in PsO and PsA patients. Advanced methods adapted from conventional techniques are of advantage to illustrate early changes in bone metabolism or quantify inflammation in the anatomical structure. With the use of these techniques, pathophysiological backgrounds of PsO and PsA are explored and can be characterized more precisely. New methods such as FOI might be able to complement other imaging techniques by quantification of changes in micro-vascularization as a very early sign of inflammation (subclinical PsA without clinical symptoms). Nevertheless, by now, it is not possible to specifically discriminate between PsO patients and those who will develop PsA by solely using imaging as a biomarker. So, further studies are needed to explore the impact of different characteristics/biomarkers (e.g., clinical data, comorbid condition, soluble/molecular biomarkers, different imaging techniques) and their impact in predicting PsA to characterize the at-risk patients precisely and explore their impact of PsO patient selection for a longitudinal follow-up to assess the development of PsA or its interception.

## Author Contributions

All authors listed have made a substantial, direct and intellectual contribution to the work, and approved it for publication.

## Conflict of Interest

The authors declare that the research was conducted in the absence of any commercial or financial relationships that could be construed as a potential conflict of interest.
